# Myopia control efficacy of individualised ocular refraction customisation spectacle lenses: A 2‐year follow‐up study

**DOI:** 10.1111/opo.70002

**Published:** 2025-08-11

**Authors:** Ji Kou, Ye Wu, Si Lei, Ling Xiong, Xiaohang Chen, Longqian Liu

**Affiliations:** ^1^ Department of Ophthalmology, West China Hospital Sichuan University Chengdu China; ^2^ Laboratory of Optometry and Vision Sciences, West China Hospital Sichuan University Chengdu China

**Keywords:** axial elongation, individualised ocular refraction customisation, myopia control

## Abstract

**Purpose:**

To assess second‐year myopia progression in children after a 1‐year randomised controlled trial of individualised ocular refraction customisation (IORC) spectacle lenses, customised to each eye's peripheral refraction.

**Methods:**

A total of 161 children were randomised to wear IORC lenses with high (+4.50 D, IORC‐H), medium (+3.50 D, IORC‐M) or low (+2.50 D, IORC‐L) myopic defocus or single‐vision lenses (SV). In year 2, all children received IORC‐H, forming the IORC‐H1/H2/H3/H4 groups, respectively. An external SV (eSV, *n* = 42) group was matched to year 2 baseline parameters. Spherical equivalent refraction (SER) and axial length (AL) were measured every 6 months.

**Results:**

Compared with the eSV group (12‐month mean [SD] changes: SER −0.56 [0.44] D, AL 0.32 [0.16] mm), all IORC‐H intervention groups demonstrated slower progression in year 2 (IORC‐H1: −0.17 [0.37] D, 0.19 [0.14] mm; IORC‐H2: −0.25 [0.41] D, 0.17 [0.16] mm; IORC‐H3: −0.24 [0.36] D, 0.17 [0.14] mm; IORC‐H4: −0.22 [0.40] D, 0.14 [0.17] mm; *p* < 0.05), with no intergroup differences (*p* > 0.05). Compared with the SV/eSV group, the IORC‐H1 group presented 2‐year reductions of 0.84 D and 0.38 mm in the SER and AL, respectively (*p* < 0.001) and exhibited minimal progression during the initial 0–6 months (*p* < 0.05), followed by constant progression over three subsequent 6‐month intervals (*p* > 0.05).

**Conclusions:**

Switching to IORC‐H in year 2 significantly reduced myopia progression and axial elongation. Maximal control efficacy occurred in the first 6 months, with sustained clinically meaningful effects thereafter.

**Key points**

- Two‐year wear of individualised customisation spectacle lenses with high myopic defocus reduced myopia progression and axial elongation.
- Peak efficacy was seen within the first 6 months and sustained clinically meaningful effects thereafter.
- Delayed initiation in year 2 achieved comparable efficacy to continuous wear.

## INTRODUCTION

The global prevalence of myopia has been increasing at an alarming rate,^1^ with emerging evidence indicating that high myopia imposes profound burdens on both the quality of life and socio‐economic systems.^2^ Crucially, even modest reductions in childhood myopia progression have been shown to reduce the risk of vision‐impairing complications in adulthood significantly.^3^ Therefore, controlling myopia progression is a critical public health priority.

Optical interventions for myopia control focus mainly on providing myopic defocus,^4^ such as through orthokeratology (OK) lenses,^5^ multifocal soft contact lenses (SCLs)^6^, ^7^ and specialised spectacle lenses.^8^, ^9^ In addition, other optical interventions are used in an attempt to control myopia through mechanisms such as reducing image contrast^10^, ^11^ and modulating aberrations.^12^, ^13^, ^14^ Recently, a clinical study revealed that inducing peripheral hyperopic defocus can also reduce myopia progression.^15^ The authors speculated that image blur,^16^ rather than defocus, might be the underlying principle of myopia control. Other researchers have proposed that both peripheral hyperopic and myopic defocus may control myopia by reducing contrast.^17^, ^18^ Pharmacological interventions, such as atropine,^19^ can be used alone or in combination with other optical interventions when necessary.^20^, ^21^ Among the various methods for myopia control, spectacle lenses are non‐invasive, relatively simple to fit, more cost‐effective^22^, ^23^ and do not involve OK‐associated infectious keratitis^24^ or side effects associated with higher concentrations of atropine.^25^ Therefore, spectacles may be a better option for a larger population.

Notably, most myopia control spectacle lenses do not provide personalised matching for the initial relative peripheral refraction (RPR) of the wearer. Whether through altering the defocus or contrast of the peripheral retinal image, the final image formed by the same lenses substantially differs across eyes because of differences in the initial RPR.^26^ This heterogeneity likely contributes to the high inter‐individual variability in myopia control efficacy, as evidenced by the correlation between the efficacy of defocus incorporated multiple segments (DIMS) lenses and baseline RPR.^27^ Specifically, DIMS lenses demonstrated poorer myopia control in children with myopic RPR compared to those with hyperopic RPR, indicating suboptimal efficacy in specific subpopulations with non‐personalised designs. This underscores the potential of individualised ocular refraction customisation (IORC) spectacle lenses (Thondar, Thondar.com). The free‐form surface^28^, ^29^ is customised based on the original RPR of each eye, which is measured using multispectral refraction topography (Thondar, Thondar.com).^30^ On this basis, microlenses distributed on the front surface of the spectacle lens are used to modify the retinal peripheral image.

In our previous 1‐year randomised controlled trial (RCT),^28^ IORC lenses with high (+4.50 D, IORC‐H) myopic defocus significantly reduced changes in spherical equivalent refraction (SER) by 0.42 D (70%) and axial length (AL) by 0.24 mm (67%) compared with single‐vision lenses (SV). However, many myopia control interventions have shown effects primarily during the first year or even in the first few months,^3^, ^31^ with a tendency towards reduced efficacy in the second year compared with the first year.^22^, ^32^ For DIMS lenses, maximal efficacy was observed within the first 6 months,^9^ whereas wearing spectacle lenses with highly aspherical lenslets (HAL) resulted in greater axial elongation in the second and third years than in the first year.^33^ These patterns highlight the insufficiency of 1‐year data for predicting long‐term IORC‐H performance^34^; thus, longer term observations are necessary. Furthermore, our previous 1‐year results demonstrated a dose–response relationship among IORC‐H and IORC lenses with medium (+3.50 D, IORC‐M) and low (+2.50 D, IORC‐L) myopic defocus, showing that higher levels of myopic defocus were associated with better myopia control efficacy. However, whether increasing the defocus magnitude by switching from medium or low to high myopic defocus can further improve myopia control efficacy remains to be evaluated.

Given that IORC‐L demonstrated negligible efficacy and that IORC‐M exhibited weaker myopia control than IORC‐H, coupled with the superior efficacy profile of IORC‐H while maintaining acceptable visual quality,^29^ all participants switched to wearing IORC‐H in this extension study. This approach simultaneously enabled continued efficacy monitoring and guaranteed that all participants received an effective intervention to safeguard participant welfare. Accordingly, this study aimed to investigate: (1) whether efficacy is sustained through two continuous years of IORC‐H wear; (2) delayed intervention effects in the original SV group that switched to wearing IORC‐H after 1 year and (3) the responses in participants switching from medium/low‐defocus IORC lenses to IORC‐H during the second year.

## METHODS

### Study design

This second‐year study, which was conducted from September 2023 to December 2024, was an extension of a 1‐year double‐blinded RCT^28^ (ChiCTR2200063036) performed at the Department of Ophthalmology, West China Hospital, Sichuan University. Briefly, during the first year, Chinese children with myopia aged 8–12 years with SERs between −4.00 D and −0.75 D were randomly assigned (1:1:1:1) to wear IORC‐H, IORC‐M, IORC‐L or SV. In year 2, participants who had completed the first‐year study were invited to continue for an additional year, during which all of the children received IORC‐H. Specifically, participants originally in the IORC‐H group continued wearing IORC‐H (IORC‐H1), whereas those initially assigned to the IORC‐M, IORC‐L or SV groups switched to IORC‐H, forming the IORC‐H2, IORC‐H3 and IORC‐H4 groups, respectively.

At the beginning of year 2, all participants underwent RPR measurements using multispectral refraction topography after cycloplegia, and new lenses were then customised on the basis of these updated RPR results. Frame adjustments were performed to ensure optimal fit for each participant. Pupil height was then measured, and the lens optical centre was aligned with the pupil. The lens replacement criteria in year 2 remained consistent with those in the first‐year protocol, requiring prescription updates if SER changes reached or exceeded 0.50 D.

The study was approved by the Biomedical Research Ethics Committee of West China Hospital, Sichuan University (No. 2021‐1191) and adhered to the tenets of the Declaration of Helsinki. Written informed consent was obtained from the participants and their guardians after a detailed explanation of the study procedures was provided.

### Study outcomes

The SER and AL were measured at baseline and at 6‐month intervals, following the same methodology as in the first‐year RCT.^28^ Cycloplegia was induced via the administration of four drops of 0.5% tropicamide (Shenyang Sinqi, sinqi.com) administered at 5‐min intervals. Subjective refraction was evaluated 20 min after the final drop according to the principle of maximum plus to maximum visual acuity (MPMVA). AL measurements were performed using a Zeiss IOL Master 700 (zeiss.com) before cycloplegia, with five consecutive measurements obtained and averaged for analysis. Participants were instructed to wear the lenses ≥12 h daily. Compliance was assessed via telephone follow‐up 1 week after year 2 lens dispensing to document the following: (1) adaptation status (yes/no), (2) time needed for adaptation (days) and (3) daily wear time (h). Follow‐ups every 3 months reinforced adherence.

### External control group

To evaluate the second‐year efficacy of continuing and switching to IORC‐H, an external single‐vision lens (eSV, *n* = 42) control group was constructed using 1:1 propensity score matching (PSM). The eSV group was specifically matched to the IORC‐H4 group (*n* = 42) rather than to other intervention groups, as only participants in the IORC‐H4 group had received no myopia control intervention in year 1, mirroring natural progression. A total of 284 participants were pooled from the SV control groups of five independent RCTs registered in the Chinese Clinical Trial Registry (ChiCTR2200064731,^31^ ChiCTR2000037443,^35^ ChiCTR2100052052, ChiCTR2100046278 and ChiCTR2100052213). All source trials were conducted between 2021 and 2023 at the same hospital and adhered to protocols identical to those used in this study for measuring SER and AL.

Owing to differences in inclusion criteria between the source RCTs and the current study (Table S1), external controls were first pre‐screened to align with the IORC‐H4 group's baseline characteristics at year 2 (age: 9–13 years; SER: −4.50 to −1.00 D; AL: 22.74–26.74 mm). A PSM with a matching tolerance of 0.01 was subsequently applied without replacement, prioritising exact matches for sex and nearest‐neighbour matches for age, SER and AL. A standard mean difference (SMD) <0.1 was considered an acceptable covariate match.

### Statistical analysis

All statistical analyses were conducted using SPSS software (version 26.0, ibm.com) and normality was assessed via Shapiro–Wilk tests. Continuous data are presented as means (standard deviations, SDs), and only right‐eye measurements were included in the analysis. The duration of daily wear across the four IORC‐H groups was compared via one‐way analysis of variance (ANOVA), and the associations between daily wear duration and changes in SER and AL were evaluated by Pearson's correlation analysis. For intragroup comparisons, paired t‐tests were applied to assess differences in myopia progression and axial elongation between year 1 and year 2 within each group. To evaluate progression across four 6‐month intervals (0–6, 6–12, 12–18 and 18–24 months) in the IORC‐H1 group, repeated‐measures ANOVA was performed to analyse temporal changes in both SER and AL. Comparisons between second‐year SER and AL changes in the four IORC‐H groups and the 1‐year changes in the eSV group were performed using generalised linear models (GLMs) adjusted for sex, baseline age and SER or AL at year 2 as covariates. Model results are reported as adjusted means with 95% confidence intervals (CIs). Post hoc analyses with Bonferroni corrections were applied, and statistical significance was defined as *p* < 0.05.

## RESULTS

### Study participants

**FIGURE 1:**
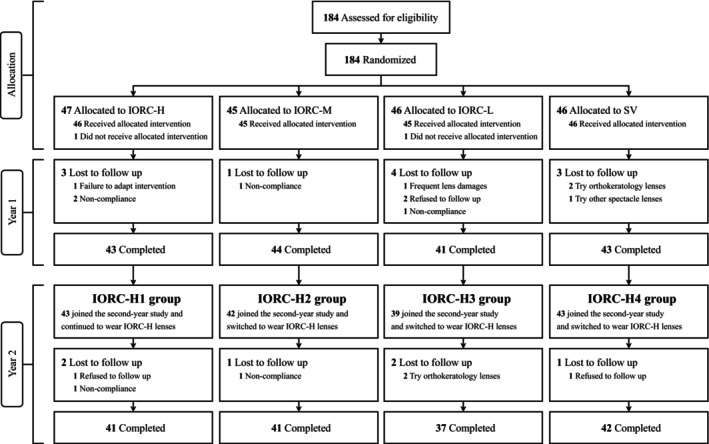
Flow chart of participants throughout the 2‐year study. IORC‐H, individualised ocular refraction customisation (IORC) lenses with high (+4.50 D) myopic defocus; IORC‐H1, children who had worn IORC‐H in year 1 and continued to wear IORC‐H in year 2; IORC‐H2, children who had worn IORC‐M in year 1 and had switched to IORC‐H in year 2; IORC‐H3, children who had worn IORC‐L in year 1 and had switched to wear IORC‐H in year 2; IORC‐H4, children who had worn SV in year 1 and had switched to wear IORC‐H in year 2; IORC‐L, IORC lenses with low (+2.50 D) myopic defocus; IORC‐M, IORC lenses with medium (+3.50 D) myopic defocus; SV, single‐vision lenses.

Figure 1 summarises participant enrolment and loss to follow‐up throughout the 2‐year study. A total of 184 children with myopia were enrolled in the initial 1‐year RCT. Among 171 participants who completed the year 1 study, 167 (98%) consented to continue into the year 2 extension phase. Ultimately, 161 children completed the 2‐year study (Table S2). The baseline characteristics of the eSV group before and after PSM are presented in Table 1. After PSM, the eSV group demonstrated a balanced baseline with the IORC‐H4 group in terms of age, sex, SER and AL (all SMDs <0.1).

**TABLE 1:** Baseline characteristics of the eSV group before and after PSM (mean [SD]).

	Before PSM			After PSM		
	IORC‐H4 (*N* = 42)	eSV (*N* = 284)	SMD	IORC‐H4 (*N* = 42)	eSV (*N* = 42)	SMD
Age (years)	10.6 (1.1)	10.3 (1.8)	0.173	10.6 (1.1)	10.6 (1.1)	<0.001
Sex (male:female)	15:27 (35.7%:64.3%)	142:142 (50%:50%)	0.290	15:27 (35.7%:64.3%)	15:27 (35.7%:64.3%)	<0.001
SER (D)	−2.60 (0.92)	−2.88 (1.47)	0.198	−2.60 (0.92)	−2.63 (0.96)	0.032
Axial length (mm)	24.68 (0.71)	24.81 (0.93)	0.144	24.68 (0.71)	24.71 (0.67)	0.043

Abbreviations: eSV, external single‐vision lenses; IORC‐H4, children who had worn single‐vision lenses in year 1 and had switched to wear individualised ocular refraction customisation lenses with high (+4.50 D) myopic defocus in year 2; PSM, propensity score matching; SER, spherical equivalent refraction; SMD, standard mean difference.

### Myopia progression in year 2

**FIGURE 2:**
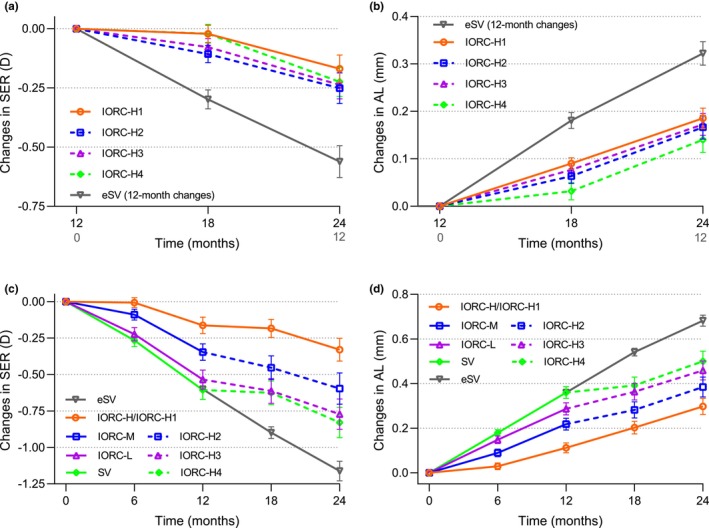
Mean (±SEM) changes in SER (a) and AL (b) in the IORC‐H1, IORC‐H2, IORC‐H3 and IORC‐H4 groups during the second year and cumulative changes in SER (c) and AL (d) from baseline to 24 months. The grey lines represent the 1‐year changes in the eSV control group. The orange lines represent changes in the IORC‐H1 group. The blue, rose, red and green dotted lines indicate the second‐year period during which participants originally assigned to IORC‐M, IORC‐L or SV switched to wearing IORC‐H. AL, axial length; eSV, external single‐vision lens; IORC‐H, individualised ocular refraction customisation (IORC) lenses with high (+4.50 D) myopic defocus; IORC‐H1, children who had worn IORC‐H in year 1 and continued to wear IORC‐H in year 2; IORC‐H2, children who had worn IORC‐M in year 1 and had switched to IORC‐H in year 2; IORC‐H3, children who had worn IORC‐L in year 1 and had switched to wear IORC‐H in year 2; IORC‐H4, children who had worn SV in year 1 and had switched to wear IORC‐H in year 2; IORC‐L, IORC lenses with low (+2.50 D) myopic defocus; IORC‐M, IORC lenses with medium (+3.50 D) myopic defocus; SER, spherical equivalent refraction; SV, single‐vision lenses.

The eSV group presented a mean SER change of −0.56 (0.44) D over a 1‐year period. During the second year, the mean SER changes were −0.17 (0.37) D, −0.25 (0.41) D, −0.24 (0.36) D and −0.22 (0.40) D in the IORC‐H1, IORC‐H2, IORC‐H3 and IORC‐H4 groups, respectively (Table 2 and Figure 2a). GLM analysis revealed that sex (95% CI: −0.306 to −0.081; *p* = 0.001) and baseline age at year 2 (95% CI: 0.041–0.137; *p* < 0.001) were significantly associated with myopia progression. The model‐adjusted SER changes during the second year revealed substantially reduced progression across all IORC‐H groups compared with the eSV group (Table 3). Specifically, compared with the eSV group, the IORC‐H1 to IORC‐H4 groups presented reductions of 0.35 D (65%, *p* < 0.001), 0.30 D (56%, *p* = 0.002), 0.30 D (56%, *p* = 0.004) and 0.33 D (61%, *p* < 0.001), respectively. Pairwise comparisons among the four IORC‐H groups revealed no statistically significant differences in SER changes (all *p* > 0.05).

**TABLE 2:** Mean (SD) and cumulative changes in SER and AL from baseline to 24 months in the IORC‐H/IORC‐H1, IORC‐M/IORC‐H2, IORC‐L/IORC‐H3 and SV/IORC‐H4 groups.

Time (months)	IORC‐H/IORC‐H1 (*n* = 41)	IORC‐M/IORC‐H2 (*n* = 41)	IORC‐L/IORC‐H3 (*n* = 37)	SV/IORC‐H4 (*n* = 42)	IORC‐H/IORC‐H1 (*n* = 41)	IORC‐M/IORC‐H2 (*n* = 41)	IORC‐L/IORC‐H3 (*n* = 37)	SV/IORC‐H4 (*n* = 42)
	SER (D)				Changes in SER (D)			
0	−2.10 (0.77)	−2.37 (0.74)	−2.36 (0.71)	−1.99 (0.74)	–	–	–	–
6	−2.10 (0.81)	−2.45 (0.83)	−2.59 (0.80)	−2.26 (0.84)	−0.01 (0.21)	−0.09 (0.24)	−0.22 (0.28)	−0.26 (0.27)
12	−2.26 (0.93)	−2.71 (0.89)	−2.90 (0.85)	−2.60 (0.92)	−0.16 (0.36)	−0.34 (0.36)	−0.53 (0.39)	−0.60 (0.42)
18	−2.28 (0.95)	−2.82 (1.01)	−2.98 (0.91)	−2.62 (0.98)	−0.18 (0.39)	−0.45 (0.52)	−0.61 (0.52)	−0.63 (0.53)
24	−2.43 (0.98)	−2.96 (1.11)	−3.14 (0.98)	−2.82 (1.07)	−0.33 (0.50)	−0.59 (0.69)	−0.77 (0.63)	−0.83 (0.67)

Time (months)	AL (mm)				Changes in AL (mm)			
0	24.38 (0.69)	24.44 (0.71)	24.72 (0.73)	24.32 (0.67)	–	–	–	–
6	24.41 (0.70)	24.53 (0.73)	24.86 (0.72)	24.50 (0.68)	0.03 (0.09)	0.09 (0.11)	0.15 (0.10)	0.18 (0.09)
12	24.49 (0.70)	24.66 (0.75)	25.00 (0.72)	24.68 (0.71)	0.11 (0.14)	0.22 (0.17)	0.29 (0.16)	0.36 (0.17)
18	24.59 (0.71)	24.72 (0.77)	25.08 (0.72)	24.71 (0.75)	0.20 (0.18)	0.28 (0.23)	0.36 (0.22)	0.39 (0.25)
24	24.68 (0.74)	24.83 (0.77)	25.18 (0.73)	24.82 (0.76)	0.30 (0.23)	0.38 (0.29)	0.46 (0.26)	0.50 (0.29)

*Note*: The grey shading indicates data collected during year 2, when participants in all groups wore IORC‐H lenses.

Abbreviations: AL, axial length; IORC‐H, individualised ocular refraction customisation (IORC) lenses with high (+4.50D) myopic defocus; IORC‐H1, children who had worn IORC‐H in year 1 and continued to wear IORC‐H in year 2; IORC‐H2, children who had worn IORC‐M in year 1 and had switched to wear IORC‐H in year 2; IORC‐H3, children who had worn IORC‐L in year 1 and had switched to wear IORC‐H in year 2; IORC‐H4, children who had worn SV in year 1 and had switched to wear IORC‐H in year 2; IORC‐L, IORC lenses with low (+2.50 D) myopic defocus; IORC‐M, IORC lenses with medium (+3.50 D) myopic defocus; SER, spherical equivalent refraction; SV, single‐vision lenses.

**TABLE 3:** Mean (95% CI) adjusted changes in SER and AL in year 2.

	IORC‐H1 (*n* = 41)	IORC‐H2 (*n* = 41)	IORC‐H 3 (*n* = 37)	IORC‐H4 (*n* = 42)	eSV (*n* = 42)	*p*
Changes in SER (D)	−0.18 (−0.30 to −0.07)	−0.24 (−0.35 to −0.12)	−0.23 (−0.36 to −0.11)	−0.21 (−0.32 to −0.09)	−0.54 (−0.65 to −0.43)	<0.001
Changes in AL (mm)	0.19 (0.15–0.24)	0.16 (0.12–0.21)	0.17 (0.12–0.21)	0.14 (0.09–0.18)	0.32 (0.27–0.36)	<0.001

*Note*: The generalised linear model was adjusted for sex, age, SER and AL at 12 months.

Abbreviations: AL, axial length; CI, confidence intervals; eSV, external single‐vision lenses; IORC‐H1, children who had worn individualised ocular refraction customisation (IORC) lenses with high (+4.50D) myopic defocus (IORC‐H) in year 1 and continued to wear IORC‐H in year 2; IORC‐H2, children who had worn IORC lenses with medium (+3.50 D) myopic defocus in year 1 and had switched to wear IORC‐H in year 2; IORC‐H3, children who had worn IORC lenses with low (+2.50 D) myopic defocus in year 1 and had switched to wear IORC‐H in year 2; IORC‐H4, children who had worn single‐vision lenses in year 1 and had switched to wear IORC‐H in year 2; SER, spherical equivalent refraction.

**FIGURE 3:**
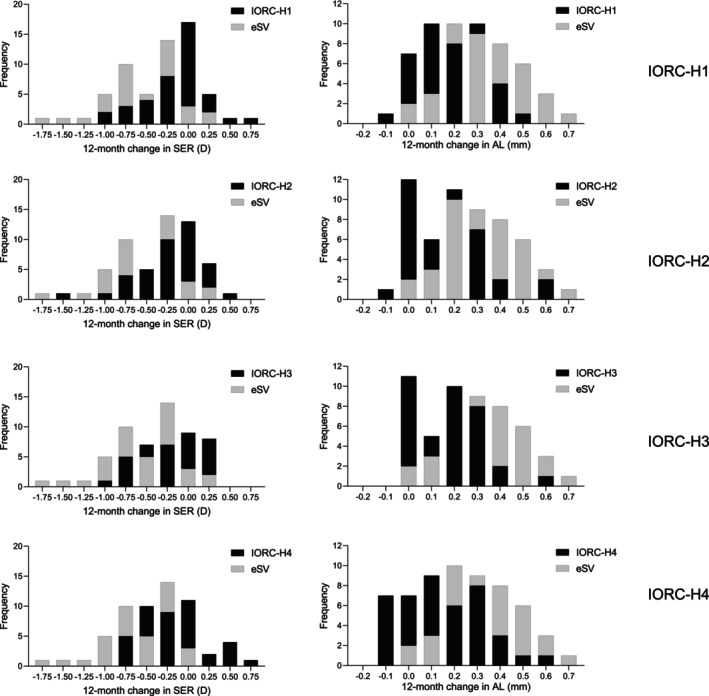
Distribution of 12‐month changes in SER and AL in the eSV group compared with the IORC‐H1, IORC‐H2, IORC‐H3 and IORC‐H4 groups during year 2. AL, axial length; eSV, external single‐vision lenses; IORC‐H1, children who had worn individualised ocular refraction customisation (IORC) lenses with high (+4.50D) myopic defocus (IORC‐H) in year 1 and continued to wear IORC‐H in year 2; IORC‐H2, children who had worn IORC lenses with medium (+3.50 D) myopic defocus in year 1 and had switched to wear IORC‐H in year 2; IORC‐H3, children who had worn IORC lenses with low (+2.50 D) myopic defocus in year 1 and had switched to wear IORC‐H in year 2; IORC‐H4, children who had worn single‐vision lenses in year 1 and had switched to wear IORC‐H in year 2; SER, spherical equivalent refraction.

The proportion of participants with no myopic shift in SER was 12% in the eSV group, 39% in IORC‐H1, 41% in IORC‐H2, 38% in IORC‐H3 and 40% in the IORC‐H4 group (Figure 3). Conversely, myopia progression exceeding 0.50 D was observed in 48%, 12%, 22%, 19% and 21% of the eSV, IORC‐H1, IORC‐H2, IORC‐H3 and IORC‐H4 groups, respectively.

### Axial elongation in year 2

The eSV group presented a mean axial elongation of 0.32 (0.16) mm over a 1‐year period. During the second year, the axial elongation was 0.19 (0.14), 0.17 (0.16), 0.17 (0.14) and 0.14 (0.17) mm in the IORC‐H1, IORC‐H2, IORC‐H3 and IORC‐H4 groups, respectively (Table 2 and Figure 2b). GLM analysis revealed significant associations between axial elongation and sex (95% CI: 0.015–0.104; *p* = 0.009) as well as baseline age at year 2 (95% CI: −0.052 to −0.015; *p* = 0.001). Compared with that in the eSV group, the model‐adjusted reduction in axial elongation was 0.12 mm (38%, *p* = 0.002) in the IORC‐H1 group, 0.15 mm (47%, *p* < 0.001) in the IORC‐H2 group, 0.15 mm (47%, *p* < 0.001) in the IORC‐H3 group and 0.18 mm (56%, *p* < 0.001) in the IORC‐H4 group (Table 3). Pairwise comparisons among the four IORC‐H groups revealed no statistically significant differences in axial elongation (all *p* > 0.05).

In the eSV group, 7% of the subjects demonstrated axial elongation of less than 0.10 mm. The corresponding proportions were 29%, 37%, 35% and 48% in the IORC‐H1, IORC‐H2, IORC‐H3 and IORC‐H4 groups, respectively (Figure 3). For axial elongation exceeding 0.30 mm, the proportions were 57% in the eSV group, 17% each in the IORC‐H1, IORC‐H2 and IORC‐H4 groups and 22% in the IORC‐H3 group.

### Changes in SER and AL in the IORC‐H1 group over 2 years

The IORC‐H1 group exhibited total myopia progression of −0.33 (0.50) D and axial elongation of 0.30 (0.23) mm over 2 years (Table 2). Compared with the SV/eSV control group, which exhibited a 2‐year myopia progression of −1.16 (0.57) D and an axial elongation of 0.68 (0.25) mm, the IORC‐H1 group exhibited a 2‐year reduction of 0.84 D (72%, *t* = 7.07, *p* < 0.001) in SER and 0.38 mm (56%, *t* = −7.22, *p* < 0.001) in AL. No significant difference was detected regarding SER changes in the IORC‐H1 group between year 1 and year 2 (*t* = −0.07, *p* = 0.94; Table 2 and Figure 2c), whereas axial elongation exhibited a statistically significant increase of 0.07 (0.16) mm in year 2 compared with year 1 (*t* = 2.98, *p* = 0.005; Figure 2d). Further analysis of progression at 6‐monthly intervals (Table S3) revealed significant time‐dependent variations for the changes in both SER (*F*_3,120_ = 4.48, *p* = 0.005) and AL (*F*_3,120_ = 8.00, *p* < 0.001). Post hoc comparisons incorporating Bonferroni correction revealed significantly slower myopia progression during the initial 6 months than during the 6–12‐month interval (*p* = 0.04), whereas axial elongation during the initial 6 months was significantly slower than that observed during 6–12 months (*p* = 0.002), 12–18 months (*p* = 0.005) or 18–24 months (*p* = 0.002). No significant differences were detected between the 6–12‐, 12–18‐ and 18–24‐month intervals for changes in either SER or AL (all *p* > 0.05).

### Changes in SER and AL over 2 years in switching groups

The IORC‐H2 group showed no significant interannual difference in SER change between year 1 and year 2 (*t* = 1.67, *p* = 0.10; Table 2 and Figure 2c), whereas the AL change decreased by 0.05 (0.16) mm in year 2 compared with year 1 (*t* = −2.04, *p* = 0.05; Figure 2d). Both the IORC‐H3 and IORC‐H4 groups exhibited reduced progression in year 2 for both SER and AL. The IORC‐H3 group demonstrated a 0.30 (0.42) D reduction in myopia progression (*t* = 4.35, *p* < 0.001) and a 0.11 (0.17) mm deceleration in axial elongation (*t* = −4.19, *p* < 0.001), whereas the IORC‐H4 group showed reductions of 0.38 (0.47) D in myopia progression (*t* = 5.26, *p* < 0.001) and 0.22 (0.18) mm in axial elongation (*t* = −7.90, *p* < 0.001).

### Daily wear duration

Among the 161 participants, 87 (54%), 49 (30%), 19 (12%) and 6 (4%) successfully adapted to the newly assigned IORC‐H at the second‐year baseline within 1, 2, 3 or 4 days of wear initiation, respectively. The mean daily wear durations for the IORC‐H1, IORC‐H2, IORC‐H3 and IORC‐H4 groups were 13.9 (1.1) h, 13.8 (0.9) h, 13.8 (0.7) h and 13.6 (1.2) h, respectively, with no significant intergroup differences (*F*_3,157_ = 0.65, *p* = 0.59). Part‐time wearing patterns (defined as daily wearing <12 h) were infrequent, occurring in one participant from the IORC‐H1 group and two participants from the IORC‐H4 group, while no cases were recorded in the IORC‐H2 or IORC‐H3 groups. No significant correlation was found between the daily wear duration and the second‐year changes in SER or AL in any of the groups (all *p* > 0.05).

## DISCUSSION

Over the 2‐year period, participants wearing IORC‐H lenses exhibited total myopia progression of −0.33 (0.50) D and axial elongation of 0.30 (0.23) mm. Notably, the participants showed minimal changes (−0.01 [0.21] D of SER and 0.03 [0.09] mm of AL) during the first 6‐monthly interval and demonstrated comparable progression across the latter three 6‐monthly intervals. Compared with the combined 2‐year changes in the first‐year SV group and second‐year eSV group, IORC‐H wear reduced the shift in SER by 0.84 D (72%) and the change in AL by 0.38 mm (56%). Additionally, participants who switched to IORC‐H in year 2—regardless of their initial assignment to the IORC‐M, IORC‐L or SV group—showed significantly reduced progression compared with the eSV group, with no significant differences observed compared with the continuous IORC‐H group during year 2. These findings suggest that delayed initiation of IORC‐H intervention remains effective.

### Usability of the eSV group

Since the original SV group switched to wearing IORC‐H in year 2, PSM was used to construct an eSV group as the control. The PSM candidate data were derived from the SV groups of RCTs conducted within the same department as this study, ensuring consistent measurement methods and instruments for both SER and AL. All SMDs were <0.1, confirming adequate comparability on the basis of the included covariates. This approach not only upheld ethical considerations, as all participants received the IORC‐H intervention in year 2, but also optimised clinical research efficiency while maximising the comparability of the eSV group.

For children with a mean baseline age of 10.6 years in year 2, the PSM‐derived eSV group showed annual changes of −0.56 D and 0.32 mm in SER and AL, respectively. Compared with the first‐year SV group results (−0.60 D in SER and 0.36 mm in AL), the expected age‐related reductions in progression during the subsequent year were 9.7% (0.06 D) for SER^36^ and 15% (0.05 mm) for AL.^37^ The observed differences (−0.04 D and 0.04 mm) were very close to the expected findings. Furthermore, progression in the eSV group was consistent with published data for Chinese children of a similar age wearing SV.^9^, ^38^ Therefore, this eSV group served as a valid and feasible control group for evaluating second‐year myopia control efficacy, allowing combined analysis with the first‐year SV group to examine 2‐year IORC‐H performance.

### Two‐year myopia control efficacy of IORC‐H

Compared with the SV/eSV group, 2‐year continuous wear of IORC‐H reduced myopia progression by 0.84 D (72%) and axial elongation by 0.38 mm (56%). This efficacy was comparable to that seen with HALs, whose use resulted in reductions of 0.80 D (55%) in SER and 0.35 mm (51%) in AL.^8^ While DIMS lenses showed comparable inhibition of axial elongation (0.34 mm, 62%),^9^ their SER control efficacy (0.44 D, 52%) was inferior to that of IORC‐H. Other interventions, including OK lenses,^5^ dual‐focus SCLs^6^ and high‐add power (+2.50 D) SCLs^39^ generally exhibited weaker control effects.

Variations in lens design,^4^ participant age,^40^ measurement protocols and progression rates in the control groups^3^ may have contributed to differences in reported efficacy across studies. Notably, the SV/eSV group exhibited slower myopia progression (−1.16 D) than the HAL study control group (−1.46 D).^8^ Less progression in the control group may reduce the calculated treatment effect, potentially underestimating the myopia control seen with IORC‐H lenses. Nevertheless, the available evidence demonstrates favourable myopia control efficacy with the use of IORC‐H. This enhanced efficacy may be attributed to the eye‐specific optical design. While animal studies have suggested that peripheral form deprivation exacerbates axial myopia,^41^ clinical evidence indicates that a reduction in peripheral retinal image quality may inhibit human myopia progression.^10^, ^11^, ^15^ Regardless of whether the mechanism involves defocus or contrast theory, IORC lenses are customised on the basis of the initial RPR to generate an expected magnitude of peripheral defocus or image blur, potentially enhancing myopia control effects.

### Temporal patterns of control efficacy

Although IORC‐H demonstrated pronounced efficacy over 2 years, progression rates were not uniform across intervals. Comparative analysis of the 6‐monthly progression in the IORC‐H1 group revealed markedly slower myopia progression during the initial 6 months, with minimal changes being observed for both SER (0.01 D) and AL (0.03 mm). A relatively consistent progression pattern subsequently emerged, with mean 6‐monthly changes of 0.11 D in SER and 0.09 mm in AL over the following 18 months.

This pattern mirrors findings for other interventions. A meta‐analysis of multifocal spectacles^34^ reported a progressive decrease in efficacy after the initial 6 months. Similarly, OK lenses^5^ and SCLs with positive spherical aberration^14^ showed maximal control efficacy within the first 6 months. Brennan et al.^3^ proposed a ‘two‐phase’ model for myopia control interventions, characterised by an initial strong inhibitory phase followed by a stabilisation phase. The current observations are in line with this model, although encouragingly, IORC‐H maintained clinically meaningful efficacy even during the latter phases.

Potential explanations for the reduction in efficacy include age‐related slowing of natural progression,^5^, ^9^, ^42^, ^43^ the choroidal response^44^ or retinal adaptation to optical signals.^5^, ^44^ Notably, compliance^7^, ^8^ remained high throughout the study, thereby ruling out a decrease in lens wearing time as a contributing factor. A unique consideration for IORC lenses involves dynamic RPR changes during myopia progression.^45^ While participants received updated lenses at 0 and 12 months on the basis of their RPR measurements, new interim prescriptions were made only for SER shifts ≥0.50 D. This contrasts with HAL protocols,^46^ employing fixed biannual lens replacements irrespective of refractive status. Notably, although all participants received updated IORC‐H at 12 months, the progression rates from 12 to 18 months remained comparable with those from 6 to 12 months and from 18 to 24 months (Figure 2c,d). Whether more frequent RPR‐guided lens updates could enhance long‐term efficacy warrants further investigation, particularly given that changes in ocular shape may counteract the initial optical characteristics.^42^ Forthcoming longitudinal analyses of RPR in our laboratory may provide further insight into this question.

### Delayed intervention effects

The first‐year RCT demonstrated a dose‐dependent relationship between the magnitude of myopic defocus and myopia control efficacy for IORC lenses.^28^ In the second‐year extension, intragroup comparisons revealed that participants switching from IORC‐M to IORC‐H in year 2 presented significantly slower axial elongation, whereas those switching from IORC‐L or SV to IORC‐H showed reductions in the changes in both SER and AL. These findings parallel observations in HAL studies,^33^ whereby switching from SAL to HAL in year 3 slowed axial elongation despite myopia progression. However, interpretation requires caution because of the inherent age‐related progression deceleration,^47^, ^48^ which may confound self‐controlled comparisons.

Intergroup analyses revealed consistent efficacy regardless of the first‐year intervention (IORC‐H, IORC‐M, IORC‐L or SV), with all groups switching to IORC‐H in year 2 and demonstrating significantly slower progression than those in the eSV group. Notably, the SV‐to‐IORC‐H group presented the characteristic ‘initial strong inhibitory phase’^3^ from 12 to 18 months (Figure 2). All delayed‐intervention groups exhibited progression comparable to that of continuous IORC‐H wearers, which is consistent with previous clinical observations.^33^, ^49^ This suggests that the year‐2 efficacy remains robust irrespective of prior exposure to IORC lenses of varying myopic defocus magnitudes. However, whether previous use of non‐IORC interventions (e.g., atropine or alternative spectacle designs) impacts IORC‐H efficacy remains unexplored.

The potential desensitisation of retinal responses to sustained optical signals^5,44^ raises questions as to whether gradually increasing the magnitude of defocus could counteract diminishing sensitivity. However, the present data demonstrate superior cumulative efficacy with immediate IORC‐H initiation compared with stepped approaches (IORC‐H2 and IORC‐H3 groups; Table 2 and Figure 2). Alternative strategies, such as pulsed IORC‐H intervention (e.g., alternating wear cycles) or increased defocus magnitudes beyond 4.50 D, warrant further investigation. However, rigorous safety assessments and visual quality evaluations must precede clinical trials of higher defocus configurations.

### Limitations

Firstly, while PSM was employed to construct the eSV control group, some confounding factors influencing myopia progression—such as near work^50^ and outdoor activity^51^—were not accounted for. Nevertheless, the analysis was restricted to datasets from a single clinical centre, employing identical SER and AL measurement protocols across almost concurrent clinical trials to enhance intergroup comparability. Moreover, the reasonable progression observed in the eSV group supports the validity of this comparative approach. Secondly, the use of subjective refraction for SER measurements, despite standardised protocols, introduces potential examiner bias, which could be reduced by objective autorefraction. However, the objectively measured AL provides robust validation of efficacy. Thirdly, the 2‐year follow‐up period remains insufficient to evaluate long‐term efficacy fully. Based on the observed stabilisation phase^3^ after the initial 6‐month period, it seems reasonable to postulate sustained efficacy of IORC‐H beyond 2 years. Nevertheless, extended follow‐up studies (>5 years) are needed to assess the sustainability of the effect. Fourthly, this study cannot predict potential rebound effects upon discontinuation of IORC‐H. While 0.1% atropine demonstrates characteristic post‐cessation rebound,^52^ existing evidence suggests minimal rebound with myopia control spectacles.^53^ Future trials should compare IORC‐H discontinuation patterns against age‐matched controls to quantify the rebound magnitude.

## CONCLUSIONS

IORC‐H spectacle lenses, featuring free‐form surfaces customised for individual peripheral refraction, maintain considerable myopia control efficacy during the second year of wear. While the maximal inhibitory effect occurred during the initial 6 months, subsequent progression remained slower than that in SV controls, with comparable progression across the latter three 6‐month intervals. Delayed initiation of the IORC‐H intervention achieved comparable second‐year efficacy to that observed with continuous IORC‐H intervention.

## AUTHOR CONTRIBUTIONS

**Ji Kou:** Conceptualization (equal); data curation (equal); formal analysis (equal); investigation (equal); methodology (equal); project administration (equal); software (equal); visualization (equal); writing – original draft (equal). **Ye Wu:** Conceptualization (equal); data curation (equal); formal analysis (equal); investigation (equal); methodology (equal); project administration (equal); resources (equal); visualization (equal); writing – original draft (equal). **Si Lei:** Data curation (equal); investigation (equal); methodology (equal); project administration (equal); visualization (equal). **Ling Xiong:** Data curation (equal); investigation (equal); methodology (equal); project administration (equal). **Xiaohang Chen:** Data curation (equal); funding acquisition (supporting); investigation (equal); software (equal). **Longqian Liu:** Conceptualization (equal); funding acquisition (lead); investigation (equal); methodology (equal); resources (equal); supervision (lead); validation (lead); writing – review and editing (lead).

## FUNDING INFORMATION

The Collaborative Research Agreement between West China Hospital, Sichuan University and Chengdu Huashi Micro‐Professional Vision Technology Company Limited, Shenzhen Thondar Technology Corporation (HX‐H2112325); and the Postdoctoral Fellowship Program of CPSF (GZC20231794).

## CONFLICT OF INTEREST STATEMENT

The authors declare that they have no conflicts of interest.

## PATIENT CONSENT STATEMENT

Written informed consent was obtained from the participants and their guardians after a detailed explanation of the study procedures was provided.

## Supporting information


Data S1

## Data Availability

The data supporting the findings of this study are available from the corresponding author Longqian Liu, upon reasonable request.
