# Supplementary material for: Myopia control efficacy of individualised ocular refraction customisation spectacle lenses: A 2‐year follow‐up study
Source: Ophthalmic Physiol Opt. 2025 Aug 11;45(7):1882–93. doi: 10.1111/opo.70002 (PMC12682103; doi:10.1111/opo.70002)
Supplement: Supplementary file 1 — Data S1 [file OPO-45-1882-s001.docx]

| **Supplemental Table 1** Inclusion criteria for age and SER in the current study and five external randomised controlled trials | | | |
| --- | --- | --- | --- |
| Registration number | Sample size of control group | Age (years) | SER (D) |
| ChiCTR2200063036 (the current study) | 42 (IORC-H4 group) | 9–13 (year 2) | −4.50 to −1.00 (year 2) |
| ChiCTR2200064731 | 30 | 8–12 | −4.00 to −0.75 |
| ChiCTR2000037443 | 79 | 6–15 | −8.00 to 0.00 |
| ChiCTR2100052052 | 65 | 6–16 | −7.00 to −0.75 |
| ChiCTR2100046278 | 60 | 8–15 | −8.00 to −0.75 |
| ChiCTR2100052213 | 50 | 6–16 | −7.50 to −0.75 |
| SER, spherical equivalent refraction; IORC-H4, children who had worn single-vision lenses in year 1 and switched to wear individualised ocular refraction customisation lenses with high (+4.50D) myopic defocus in year 2 | | | |

| **Supplemental Table 2** Year 2 baseline characteristics of participants completing the 2-year follow-up (mean [SD]) | | | | |
| --- | --- | --- | --- | --- |
|  | IORC-H1  (n=41) | IORC-H2  (n=41) | IORC-H 3  (n=37) | IORC-H4  (n=42) |
| Age (years) | 10.3 (1.0) | 10.5 (1.1) | 10.4 (1.1) | 10.6 (1.1) |
| Sex (male:female) | 21:20 | 17:24 | 25:12 | 15:27 |
| Cycloplegic SER (D) | −2.26 (0.93) | −2.71 (0.89) | −2.90 (0.85) | −2.60 (0.92) |
| Axial length (mm) | 24.49 (0.70) | 24.66 (0.75) | 25.00 (0.72) | 24.68 (0.71) |
| IORC-H1, children who had worn individualised ocular refraction customisation (IORC) lenses with high (+4.50D) myopic defocus (IORC-H) in year 1 and continued to wear IORC-H in year 2; IORC-H2, children who had worn IORC lenses with medium (+3.50 D) myopic defocus in year 1 and had switched to wear IORC-H in year 2; IORC-H3, children who had worn IORC lenses with low (+2.50 D) myopic defocus in year 1 and had switched to wear IORC-H in year 2; IORC-H4, children who had worn single-vision lenses in year 1 and had switched to wear IORC-H in year 2; SER, spherical equivalent refraction. | | | | |

| **Supplemental Table 3** Mean (SD) changes in SER and AL over 6-month intervals in the IORC-H1 group | | | | |
| --- | --- | --- | --- | --- |
| Interval | Changes in SER (D) | Comparisons p-values (0–6 vs. 6–12; 0–6 vs. 12–18; 0–6 vs. 18–24; 6–12 vs. 12–18; 6–12 vs. 18–24; 12–18 vs. 18–24 months) ^†^ | Changes in AL (mm) | Comparisons p-values (0–6 vs. 6–12; 0–6 vs. 12–18; 0–6 vs. 18–24; 6–12 vs. 12–18; 6–12 vs. 18–24; 12–18 vs. 18–24 months) ^†^ |
| 0–6 months | −0.01 (0.21) | 0.04; > 0.99; 0.05; 0.14; > 0.99; 0.08 | 0.03 (0.09) | 0.002; 0.005; 0.002; > 0.99; > 0.99; > 0.99 |
| 6–12 months | −0.16 (0.27) |  | 0.08 (0.07) |  |
| 12–18 months | −0.02 (0.23) |  | 0.09 (0.08) |  |
| 18–24 months | −0.15 (0.25) |  | 0.10 (0.09) |  |
| SER, spherical equivalent refraction; AL, axial length; IORC-H1, children who had worn individualised ocular refraction customisation (IORC) lenses with high (+4.50D) myopic defocus (IORC-H) in year 1 and continued to wear IORC-H in year 2.  ^†^Following a repeated-measures ANOVA, post-hoc pairwise comparisons between intervals were performed using the Bonferroni correction. | | | | |
